# How Do Nurses Cope with Shift Work? A Qualitative Analysis of Open-Ended Responses from a Survey of Nurses

**DOI:** 10.3390/ijerph16203821

**Published:** 2019-10-10

**Authors:** Michael Savic, Rowan P. Ogeil, Megan J. Sechtig, Peta Lee-Tobin, Nyssa Ferguson, Dan I. Lubman

**Affiliations:** 1 Eastern Health Clinical School, Monash University, Box Hill, VIC 3128, Australia; michaels@turningpoint.org.au (M.S.); pleetobin@gmail.com (P.L.-T.); Dan.Lubman@monash.edu (D.I.L.); 2 Turning Point, Eastern Health, Richmond, VIC 3121, Australia; Megan.Sechtig@turningpoint.org.au (M.J.S.); NyssaF@turningpoint.org.au (N.F.); 3 Monash Addiction Research Centre, Frankston, VIC 3199, Australia

**Keywords:** shift work, stress, sleep loss, exercise/physical activity, nurses, coping, health, wellbeing

## Abstract

Nurses are frequently required to engage in shift work given the 24/7 nature of modern healthcare provision. Despite the health and wellbeing costs associated with shift work, little is known about the types of coping strategies employed by nurses. It may be important for nurses to adopt strategies to cope with shift work in order to prevent burnout, maintain wellbeing, and ensure high quality care to patients. This paper explores common strategies employed by nurses to cope with shift work. A workforce survey was completed by 449 shift working nurses that were recruited from a major metropolitan health service in Melbourne, Australia. Responses to open-ended questions about coping strategies were analysed using the framework approach to thematic analysis. Four interconnected main themes emerged from the data: (i) health practices, (ii) social and leisure, (iii) cognitive coping strategies, and (iv) work-related coping strategies. Although a range of coping strategies were identified, sleep difficulties often hindered the effective use of coping strategies, potentially exacerbating poor health outcomes. Findings suggest that in addition to improving nurses’ abilities to employ effective coping strategies on an individual level, workplaces also play an important role in facilitating nurses’ wellbeing.

## 1. Introduction

Work hours that fall outside the traditional 9am–5pm schedule are increasingly prevalent because of increased demand for services across the 24 hour day [1]. Many professions require a shift working roster to meet community need or workload, such as law enforcement, manufacturing and healthcare [2]. Approximately 1.4 million Australians are classified as shift workers, and collectively account for 16% of the total employed population [3]. Similar figures are seen internationally, with shift workers comprising approximately 15% of full-time workers in the United States [4] and 17% in the United Kingdom [5], whereas shift workers account for an estimated 28% of all working adults in Canada [6].

Shift work is associated with a series of potentially negative physical and psychological effects, as human physiology has biologically adapted to synchronise with the light–dark cycle [7]. Underlying homeostatic circadian rhythms are pervasive across many physiological and biochemical outputs [8,9]. In addition, circadian rhythmicity underlies performance and behavioural outputs including sleep–wake states, alertness, and mental performance [10,11,12].

The most commonly reported adverse effects of shift work are excessive sleepiness [13], chronic sleep disturbances including reduced total sleep time and shorter durations of stage 2 and rapid eye movement sleep [14], and insomnia [15,16]. In relation to other psychological consequences of shift work, a recent meta-analysis highlighted a significant association between poor sleep and a heightened risk of depression [17]. Shift workers are also at risk of shift work disorder, which is associated with chronic disruptions to sleep–wake schedules [18,19], and excessive sleepiness that impacts upon their wellbeing and performance [20]. The excessive sleepiness that shift workers experience, due to associated cognitive impairments, has also been linked to an increase in road and occupational accidents [14].

Adverse physical effects of shift work include increased risk of metabolic diseases (e.g., type 2 diabetes and weight gain), cardiovascular diseases (e.g., coronary heart disease), and stroke [14,21]. Since 2007, the World Health Organization has classified shift work with circadian disruption as a probable human carcinogen [22,23]. This association has been demonstrated in relation to the risk of breast cancer, with every 5 years of shift work increasing breast cancer risk by 3% [24,25].

Other research studies have reported an increased propensity for errors to be made at work by those working outside of traditional 9am–5pm hours [26]. These performance decrements may come at a cost to patients in health settings and contribute to poor quality of care [27], and/or increased likelihood of medication errors [28,29]. Furthermore, the occupational stress healthcare workers face from shift work can impact them personally, resulting in increased rates of absenteeism [30]. Collectively, these health and performance impacts highlight the importance for healthcare workers to adopt strategies to cope with the demands of shift work in order to prevent burnout, maintain wellbeing, and ensure continued high quality care for patients [31].

Lazarus and Folkman [32] theorised that coping could be categorized as either problem-focused (e.g., by employing problem solving and time management strategies) or emotion-focused (e.g., through mindfulness, relaxation, and obtaining emotional support from colleagues or friends). In the context of healthcare, the ability to cope with stressors has been linked to improved mental health [33,34]. However, avoidance coping (a potentially negative example of emotion-focused coping) with the use of alcohol and other drugs is commonly reported among shift workers, and can be detrimental to one’s health [32,33,35].

Although nurses comprise over 56% of the healthcare workforce [36], only a small body of research has explored the types of coping strategies used by nurses to manage job stress and the adverse effects of shift work. Given the prevalence of sleep disturbances associated with shift work, nurses frequently report the use of coping strategies aimed at facilitating better quality and quantity of sleep, such as installing light-darkening shades, using telephone answering machines, and white noise [37]. Other coping strategies identified among nurses include seeking social support from peers and co-workers [38,39] and planful-problem solving [33,40,41]. These practices have been found to effectively reduce stress among nurses through the use of positive outlets to debrief during and outside of work hours, and through considering strategies to practically reduce stressors [33,41]. Accounts of health coping practices such as exercise and healthy eating also feature amongst nursing samples [37,42]; however, excessive sleepiness associated with shift work schedules often hinders engagement [34,43]. A number of potentially less productive avoidance strategies used by nurses have been identified, including social distancing [44,45] and substance use [31]. Nurses’ reports of relying on medication and alcohol to aid sleep, along with caffeine to maintain alertness while on shift [31,37,46,47], may be concerning when considering the identified link with poorer sleep quality and increased psychological distress [48,49]. Such avoidance strategies focus on avoiding the stressor or the response to it, potentially exacerbating health problems [32,35].

Although the small body of existing literature has provided useful insights into the frequency of types of coping strategies employed by nurses and their effects on health outcomes, many of these studies employ quantitative approaches, limiting coping strategies to those that nurses select from pre-determined items [50,51,52]. Additionally, numerous studies have focused on nurses’ coping strategies generally, rather than in relation to shift working schedules [38,40,44,50]. Therefore, this study aimed to explore the common coping strategies used by shift working nurses in their own terms.

## 2. Materials and Methods

This article reports on the qualitative analysis of open-ended items about coping with shift work that were included in a workforce survey (given to shift workers and non-shift workers) as part of a broader study of shift worker wellbeing. Results of the quantitative analysis are reported elsewhere.

### 2.1. Participant Recruitment

Participants were recruited from a major metropolitan health service that oversees three major hospitals and multiple outpatient clinics and services in Melbourne, Australia. Participants were invited to complete a workforce survey approved by the Eastern Health Human Research Ethics Committee via e-mail messages and via employee newsletters and payslips sent by the human resources department and senior management. A generic hospital email address was used to send the email invitation in order to minimise any perceived coercion to participate from senior management and/or perceived personal relationships. Participants were provided with a written description of the project, the contact details of the researchers, and a hyperlink to the online survey prior to beginning the survey. There was no direct incentive to complete the survey; however, upon completion participants were entered into a prize draw to win one of ten gift vouchers (worth AU $100 each).

### 2.2. Participant Characteristics

The sample consisted of 449 shift working nurses. The mean age was 41 years (*SD* = 12.4). Table 1 shows the sociodemographic and occupational characteristics of the sample.

### 2.3. Data Collection

Data were collected using a workforce survey emailed to all employees of a major metropolitan health service. The survey included open-ended qualitative questions which asked participants “How has shift work impacted on your overall wellbeing (either positively or negatively)?” and “How do you cope with the demands of shift work? What do you do to maintain wellbeing?” Participants’ responses to open-opened questions ranged from a sentence to a paragraph of text. The survey also included quantitative measures of physical health, physical activity engagement and preferences, diet and nutrition, health and wellbeing, substance use, sleep, work roles, and demographics. The survey was available online and took 10–15 min to complete.

### 2.4. Data Analysis

The framework approach to thematic analysis [53] was used to qualitatively analyse open-ended survey responses. The framework approach is considered appropriate for applied and practice-relevant research, such as this study, given that it encourages themes to emerge inductively as well as being deductively derived from the study aims [53,54]. It is also a fruitful approach for the qualitative analysis of open-ended survey responses [55,56,57]. Initial coding of a number of responses into themes was conducted by the first two authors. On the basis of the authors’ knowledge of the literature and discussion, additional themes were added to the framework, and a final thematic framework was agreed upon [53]. Employing the thematic framework, the first author coded further responses, and all authors discussed interpretation of the data further to finalise the analysis.

## 3. Results

**Figure 1:**
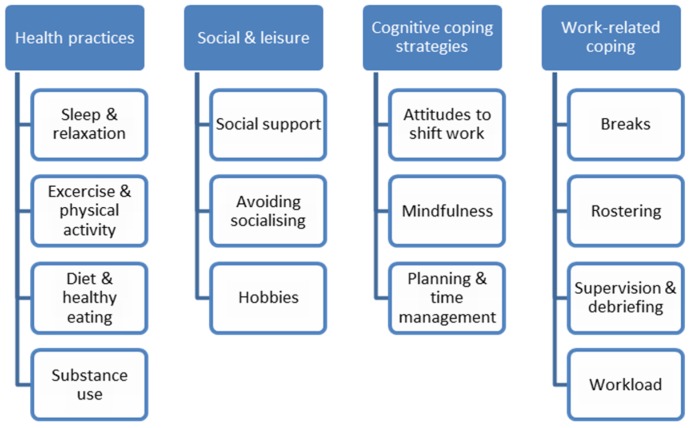
Themes and sub-themes identified.

Four main themes relating to participants’ coping strategies were identified, three of which were personal coping strategies: health practices, social and leisure, and cognitive coping strategies, while work-related coping also emerged as a theme (see Figure 1). The themes were interconnected and each comprised sub-themes involving both what may be considered positive and negative coping strategies. As will be illustrated throughout the description of themes, adopting one coping strategy often had implications for the adoption of other coping strategies.

### 3.1. Health Practices

Participants recounted the use of a number of health-related coping strategies, including sleep and relaxation, physical activity, healthy eating and diet, and substance use.

#### 3.1.1. Sleep and Relaxation

Many participants reported that a major challenge of shift work is tiredness and exhaustion. Given this, and as illustrated in the following quote, sleep and relaxation practices were amongst the most commonly reported coping strategies, and were often prioritised:

*“Try and get as much sleep as I can to allow me to be less tired.”*.

(Participant 102, female, age 25)

This comment illustrates that ensuring an adequate amount of sleep is obtained is a vital coping mechanism to maintain functioning and wellbeing. However, for some participants, sleeping came at the expense of other coping strategies:

*“I try to ensure I get rest after work but resting/sleeping affects my ability to socialise with people.”*.

(Participant 13, female, age 24)

*“I try to relax when I have days off. However, this impacts on the other activities which promote a healthy life, such as exercise and healthy eating.”*.

(Participant 253, male, age 31)

These quotes suggest that it is not simply a matter of getting more sleep or rest, as achieving that might also impede the ability to socialise with others, another coping strategy in itself. Accounts of sleep and relaxation strategies were also often interconnected with other health practices, such as diet and exercise and physical activity.

#### 3.1.2. Exercise and Physical Activity

Exercise and physical activity were common coping strategies amongst the sample, as one participant reported:

*“I de-stress through physical activity. However, when exhausted I cannot do this and this has a negative impact on my wellbeing.”*.

(Participant 307, female, age 33)

Another participant also described attempts at exercising:

*“I struggle but try and participate in exercise every day.”*.

(Participant 267, female, age 28)

These quotes highlight the benefits of exercising to reduce stress and maintain health, but also how tiredness and sleep deprivation—common adverse effects of shift work—can act as barriers to engaging in physical activity. Physical activity was often mentioned in connection to socialising, sleep, and healthy eating coping practices.

#### 3.1.3. Diet and Healthy Eating

Diet and healthy eating practices were commonly reported strategies and manifested in various ways. For example, some participants explained:

*“Learning to adapt. Eat healthy. Eat minimal on night shift and only light things such as cut up carrot.”*.

(Participant 221, female, age not specified)

*“Eat well at work. Take a full dinner break and not talk at dinner.”*.

(Participant 350, female, age 34)

Another participant highlighted the need for meal planning:

*“I pre-prepare food and shopping before my nights in order to minimise tasks and improve eating when tired.”*.

(Participant 13, female, age 24)

These comments illustrate how eating practices and ensuring a healthy diet can act as coping mechanisms while on shift, and how tiredness or exhaustion can necessitate pre-planning meals and time management to ensure healthy eating. Similar to diet and healthy eating practices, substance use was also mentioned as a strategy to combat tiredness and facilitate coping on and off shift.

#### 3.1.4. Substance Use

Accounts of using substances, such as alcohol or sleep medication, to cope with shift work manifested as both positive and negative coping mechanisms amongst the sample. Some participants identified caffeine use as an aid to cope with the challenges of maintaining wakefulness during shifts, as well as the use of prescription medication to attain quality sleep. For example, one participant explained:

*“Unfortunately falling into the quick fixes of caffeine drinks to stay awake and having to see a doctor about sleep and prescribed melatonin and temazepam.”*.

(Participant 231, female, age 34)

However, other participants described extra-medical use of medications:

*“Take a lot of sleeping tablets to at least get some sleep during the day.”*.

(Participant 435, male, age 49)

*“Take non-prescription medication and sleep.”*.

(Participant 403, male, age not specified)

Although prescribed medication can help improve sleep, caffeine and both prescription and extra-medical pharmaceutical use were considered non-desirable methods of coping in these accounts, suggesting that alternative strategies are needed to better adapt to and manage the adverse health effects of shift work. However, many participants also mentioned substance use as positive coping strategy:

*“Plan nice days off and drink alcohol.”*.

(Participant 188, female, age 27)

This example attributes substance use as a mechanism to facilitate relaxation, pleasure, and socialising outside of work. However, it was not simply the psychoactive effects of substances that enabled participants to cope. Rather, substance use, particularly alcohol use, was often reported in combination with engaging in social and leisure activities outside of work.

### 3.2. Social and Leisure

Social and leisure activities emerged as a common theme for managing the demands of shift work. Sub-themes included accounts of social support, avoiding socialising, and using hobbies as coping mechanisms.

#### 3.2.1. Social Support

Some participants described coping through receiving social support from family, friends, co-workers, and pets, across a diverse range of situations. For instance, one participant said:

*“I am a very social person and find that taking solace in my large community of friends keeps me going. We spend what time we can together, and I like to go out dancing etc. to blow off steam.”*.

(Participant 326, non-binary/third gender, age 25)

Another participant described the emotional support socialising offers:

*“I try to spend a lot of my free time socialising with friends and debriefing about anything stressful.”*.

(Participant 274, female, age 28)

These quotes underscore the importance and benefits of social supports for participants, which can help to de-stress from shift work and foster wellbeing. However, not all participants found socialising to be an effective coping strategy.

#### 3.2.2. Avoiding Socialising

While many participants found social support helpful, a small group of participants reported preferring to avoid socialising and actively sought to disconnect from others outside of work hours. For example, some participants said:

*“I like to get up as late as possible (after a shift). Withdraw to myself to get emotional/psychological recharge from a reduction in the social stimulus. I need wind down time after shifts of 2+ hours to stop mind racing and allow for some sleep.”*.

(Participant 353, male, age 43)

*“Try to engage in activities that I think are important and avoid others.”*.

(Participant 67, female, age 62)

*“I cope by being disengaged from activities requiring regular commitment. I prepare all significant others in my world for the likelihood of being tired and unable to participate in some events.”*.

(Participant 319, female, age 54)

Here, the participants emphasised the high demands and psychological effects of nursing shift work, and described the need to avoid socialising outside of work hours in order to recharge. As personal needs may differ, avoiding socialising for some may help maintain their wellbeing. However, without social connection, these participants could be missing out on a vital coping outlet that protects against the adverse effects of shift work. In addition to social-related practices, some participants considered participating in other activities, such as hobbies, as coping mechanisms.

#### 3.2.3. Hobbies

Participants described a range of interests and hobbies undertaken to cope with shift work, with gardening and yoga commonly reported, as well as a number of social-related hobbies. Some participants explained:

*“I ride my horse 3 to 4 times a week. I book annual leave so that I can participate in clinics and competitions with my horse.”*.

(Participant 271, female, age 37)

*“Try to enjoy things that provide some emotional wellbeing like gardening for me, walking dog, enjoying family, and grandchildren.”*.

(Participant 68, female, age 55)

As illustrated in these examples, participating in hobbies appears to provide enjoyment, relaxation, and opportunities to connect with other people or animals, in turn managing work-related stress and fostering wellbeing. Accordingly, attempting to organise workplace leave to facilitate participating in hobbies was common amongst participants. Likewise, allocating time to engage in more cognitive-focused practices also emerged as a theme.

### 3.3. Cognitive Coping Strategies

The use of cognitive coping strategies were commonly reported ways of coping with the demands of shift work. Sub-themes included mindfulness and spiritual practices, attitudes towards shift work, and planning and time management, whereby participants’ employed positive thought and planning processes to facilitate wellbeing and reduce stress.

#### 3.3.1. Mindfulness

Mindfulness and spiritual practices were popular coping strategies in this sample. Mindfulness refers to the mind being fully attentive to what is presently happening for an individual, what they are doing, and the environment they are responding to. As one participant said:

*“Practice mindfulness meditation daily.”*.

(Participant 184, female, age 46)

Spiritual practices, such as attending church, singing in church choir, and praying, were also employed by some, with one participant reporting:

*“Making sure l attend church, pray on the way to work, and outside activities.”*.

(Participant 211, male, age 58)

These accounts illustrate how spiritual and mindfulness practices can manage or deflect concern away from stressors associated with shift work, benefiting wellbeing. Similarly, personal perspectives or attitudes towards shift work also related to participants’ coping.

#### 3.3.2. Attitudes to Shift Work

Attitudes towards shift work emerged as a common coping strategy, which manifested in various ways. Some participants reported positive attitudes towards shift work, emphasising perceived benefits, as some participants described:

*“I like shift work as it allows me to do more activity than a regular 9–5 worker.”*.

(Participant 279, female, age 53)

*“Acknowledge I have a stable job with sustainable income, I get more time with my young family.”*.

(Participant 446, male, age 26)

In these accounts, the participants focused on the benefits that shift work offers, which potentially enhances their ability and motivation to cope effectively. However, more commonly, some participants conveyed a stoic attitude, in which they simply accepted the nature of their work and hours without complaint. For instance, some participants said:

*“I don’t feel I have any coping mechanisms, I don’t believe I need any, work is work.”*.

(Participant 306, female, age 22)

*“I have to accept it and survive.”*.

(Participant 282, female, age 62)

*“I have got used to feeling like this as done shift work for 30 years.”*.

(Participant 451, female, age 50)

The first exemplar suggests a sense of indifference or lack of concern with work conditions. This might reflect a belief that related circumstances and stressors are out of an individual’s control, as highlighted in exemplars two and three. These participants talked about simply accepting or getting used to shift work and thus did not employ other coping strategies to reduce adverse effects. Alternatively, stoicism may be useful for some, enabling them to avoid being preoccupied with, or affected by, the negative impacts of shift work.

#### 3.3.3. Planning and Time Management

As mentioned previously in relation to other themes (heath practices, social and leisure) and sub-themes (organisation of diet, managing sleep, organising social life), planning and time management coping strategies were described frequently by participants. For some participants this meant practices in relation to work:

*“I set alarms and have my roster written on the fridge. Keep a diary also and have my roster on my phone.”*.

(Participant 102, female, age 25)

For others, this also related to planning and organising time for social connection, hobbies, and health behaviours such as healthy eating and physical activity. For instance, one participant explained:

*“Making sure time is allocated to enjoy yourself.”*.

(Participant 375, female, age 30)

These comments demonstrate the use of time management and planning practices to balance life in a way that shift work might otherwise hinder, such as engagement in healthy behaviours like socialising and exercising. Participants also highlighted the importance of being organised in relation to work, involving the use of a work roster at home to help with the demands of shift work. In these instances, the role of workplaces to facilitate personal coping strategies was often identified by participants.

### 3.4. Work-Related Coping

In addition to the aforementioned coping strategies that involve shift workers taking responsibility for implementing these themselves, a number of other beneficial strategies were reported that revolved around the workplace taking responsibility. These strategies included the use of breaks, rostering, workload, and supervision and debriefing.

#### 3.4.1. Breaks

Breaks were identified as a necessary strategy for participants to cope with the psychological and physical demands of shift work. Participants often reported that they initiated these breaks, but required approval from their workplace for them to occur. Many participants emphasised the importance of breaks, both in terms of days off from work or holidays, but also during shifts. As one participant said:

*“I will utilise sick leave if exhaustion becomes overwhelming, or if my mental health feels unstable.”*.

(Participant 277, female, age not specified)

*“Try to take short A/L (annual leave) breaks to rest and recover every 3 months”*.

(Participant 436, male, age 46)

Another participant also talked about breaks during shifts, saying:

*“When I am on day shift I tend to break up my shifts to not work long stretches, if possible.”*.

(Participant 16, female, age 56)

*“Take a full dinner break.”*.

(Participant 350, female, age 34)

In both of these accounts, participants were actively involved in deciding when and how to take breaks in order to get the most out of them and maintain wellbeing. These comments highlight the critical role of the workplace to allow workers’ requests for breaks.

#### 3.4.2. Rostering

Similarly, the ability to have some level of control over individuals’ own rosters was a vital coping strategy for many participants, which also rests on organisations granting rostering preferences. For example, one participant said:

*“I ask to have my days off together because I need the break to recover.”*.

(Participant 152, female, age 60)

Similarly, other participants described:

*“I rely on good rostering so I can have at least two days off in a row after Night shift and minimal number of Late/Early short changeover occasions, work no more than 32 hours per week for past 20 years.”*.

(Participant 185, female, age 60)

*“I try to ensure I put in roster requests that suit my lifestyle to try and have as much as a normal work life balance as I can.”*.

(Participant 259, female, age 28)

These quotes underscore participants’ reliance on workplaces to grant “good rostering” in order to prevent exhaustion and maintain health and wellbeing. Some nurses may not be granted “good rostering”, suggesting this is an area that organisations can improve. While some participants relied on rostering to reduce the toll of shift work, others mentioned reducing their workload entirely as a mechanism to cope.

#### 3.4.3. Workload

While not necessarily available to all nurses, some participants reported that working a reduced workload (i.e., part-time or causal work) instead of full-time work was beneficial. As some participants explained:

*“I have reduced my hours to a 0.7 EFT [equivalent full time] commitment in one location, and add to those hours casually elsewhere (on days I am not tired). The past 12 months this has been a significant improvement in my energy levels, and I have joined a gym and trained more.”*.

(Participant 13, female, age 24)

*“Dropped my work hours to 48 hours for fortnight, trying to look after myself first rather than earning more money.”*.

(Participant 404, female, age 32)

Many participants emphasised the benefits of casual work:

*“I stress less now I have become a casual worker and have the choice of where and when to work. No worries about getting holiday leave approved.”*.

(Participant 320, female, age 59)

In these examples, the participants described reducing their workloads, in turn allowing time for engagement in other coping strategies, such as exercise and relaxation. This suggests that reducing one’s workload can help reduce the severity of the adverse effects of shift work, and maintain health and wellbeing.

#### 3.4.4. Supervision and Debriefing

Professional supervision and more informal debriefing between colleagues were also commonly reported coping strategies. Supervision and debriefing is known as a reflective time where individuals examine their professional experiences with a supervisor who is more qualified and/or experienced, or with colleagues. This may include discussing situations that caused distress or discussing ways to further develop skills and knowledge in a professional way:

*“I engage in clinical supervision.”*.

(Participant 117, male, age 27)

Many participants valued the time and space that supervision and debriefing afforded:

*“I tend to debrief at work if required before I go home, even if it means staying late, I always relax more quickly after work when I do this.”*.

(Participant 185, female, age 60)

As described in the above accounts, debriefing and supervision were described as cathartic processes, enabling shift workers to create boundaries between their work and personal life. Accordingly, work-related coping strategies often rely on workplaces and organisations to be supportive in order to encourage wellbeing.

## 4. Discussion

This study provides an exploration of the common types of coping strategies used by nurses. Four main interconnected themes were identified: health practices, social and leisure, cognitive coping strategies, and work-related coping. The findings outline a range of strategies employed, emphasising the need to improve and facilitate shift working nurses’ ability to adopt effective coping mechanisms, in order to handle the high stress associated with nursing work and a shift working schedule.

In the first theme—health practices—nurses reported using a range of relaxation and sleep, eating, physical activity, and substance use practices to cope with shift work. Consistent with past literature, sleep and relaxation practices appeared to be some of the most frequently employed and prioritised strategies utilised to address the exhaustion and tiredness associated with shift work [31,58]. Likewise, nurses’ management of their eating habits through restricting portion sizes, monitoring patterns, and pre-preparing meals also reflects previous findings in which nurses working night-shift reported similar behaviours to aid digestion and alertness [31,37,43]. As noted, many nurses attributed the adverse effects of shift work—primarily exhaustion—to thwart exercising and healthy eating attempts. This supports existing evidence describing how health-related coping practices are often hindered by the disruption of one’s circadian rhythm because of shift work [34,43].

A recent national survey of Australian adults [59] indicated almost 20% used alcohol as a sleep aid during the past month, some to quite high levels. However, accounts of alcohol use to aid sleep were not mentioned amongst this sample; rather, health behaviours such as keeping to a consistent sleep routine, exercise and keeping active, socialising, and healthy eating were more common. Gifkins et al. [31] only found alcohol consumption as a common strategy amongst a sub-sample of experienced nurses with a mean experience level of 20 years, perhaps explaining differences between the studies. Some of the lesser and perhaps more negative coping strategies mentioned were caffeine use and medications including temazepam. A past study found approximately 61% of nurses sampled to have used a sleep aid, predominately prescription medications, during the last month [46]. Use of sleep-promoting drugs has been linked to a higher risk of poorer sleep quality [49], suggesting that despite the use of medication, sleep problems and associated consequences may persist. Caffeine use to aid alertness during shift work was also common amongst nurses in other studies [47,60,61]; however, it was not necessarily an effective coping mechanism. While moderate caffeine doses up to 200 mg (daily limit of 400 mg) can increase alertness and improve cognitive performance, higher doses can often result in poorer sleep outcomes [48,62]. Substance use, including excessive caffeine use, can be a means of avoidance coping, which often does not effectively address stressors and is associated with negative health outcomes [34,35,48]. Although only a small group of participants reported substance use strategies, considering the potentially harmful consequences, it is still of concern for shift workers’ physical and psychological health.

In the second theme—social and leisure activities—social support, avoiding socialising, and participating in hobbies emerged as common coping strategies. Consistent with findings from previous studies, social support was one of the most commonly mentioned strategies utilised to reduce stress and maintain wellbeing [33,40,63,64]. Socialising and receiving social support has been consistently associated with improved wellbeing and satisfaction with shift work [33,34,65]. A review and meta-analysis of studies on factors associated with mortality found that having adequate social supports had similar benefits in terms of preventing mortality as stopping smoking [66]. Social connection is thought to provide opportunities for social, emotional, and practical support, as well as providing a sense of belonging and identity, which can act as a resource to cope with challenging life events and situations, potentially including shift work [67]. Hobbies were also mentioned as an outlet to reduce stress. Participating in hobbies can improve shift workers’ health and wellbeing, and reduce stress through satisfying personal needs such as creativity and achievement [34,65].

Although accounts of social support strategies are prevalent across previous studies [34,40,63], a small group of nurses in the current study reported avoiding socialising as a coping mechanism. Social isolation is common amongst nurses because of shift work schedules [68], and often thwarts stress reduction practices, exacerbating health problems [33,34]. In studies conducted by Dorrian et al. [45] and Happell et al. [44], nurses also reported avoiding social interaction outside of work as a coping mechanism. However, this was stated to have a negative psychological impact upon the shift workers and their family members. Happell et al. [44] concluded that avoiding others might be a maladaptive coping response due to being too emotionally and physically exhausted to socialise, potentially contributing to adverse health and wellbeing outcomes associated with limited social support. However, other studies have suggested socially distancing oneself from others to be beneficial for some nurses in handling stress, as it allows for opportunity for reflection and problem-solving [33,52]. These contrasting social coping strategies may reflect personality differences, as broader literature has illustrated introverted individuals to be less likely to seek social support in order to cope with stressors [69]. Likewise, introverted nurses often do not require as much work-related peer support to avoid emotional exhaustion or burnout [70]. Given these differences, further investigation into the effect of personality traits could benefit understandings of the implications of avoiding socialising.

In the third theme—cognitive coping strategies—participants reported a range of mindfulness and spiritual practices, attitudes towards shift work, and time management strategies to help cope with shift work. Notably, many participants reported use of mindfulness techniques to cope, which involve focusing attention on the present moment rather than ruminating over stressors. Along with the use of similar spiritual rituals, use of these techniques corresponds with past studies, which found subsequent reductions in stress amongst nurses [61,71]. Accordingly, Grover et al. [72] applied the job demands-resources model (JD-R, Schaufeli and Taris 2014) to test the paths in which mindfulness can reduce stress across a sample of Australian nurses. Grover et al. [72] concluded that nurses who are mindful have a greater understanding of emotional demands and their effect on themselves, and thus tend to experience lower emotional demands. Given, the benefits of mindfulness practices, education and intervention programs may help nurses to reduce work-related stress.

Participants’ attitudes towards shift work also emerged as a type of cognitive coping strategy, varying from positive to stoic attitudes. Positive attitudes towards shift work amongst nurses have been associated with reduced stress, greater job satisfaction, and positive health outcomes [34,52,73]. However, more commonly conveyed within this sample was a stoic attitude, suggesting a lack of concern about shift work conditions and impacts, dismissing the need for coping strategies. Consistent with past studies [38], stoicism among nurses may be an avoidance coping strategy whereby nurses avoid thinking about stressors. Some past studies have suggested that nurses who avoid thinking about the problem often hold beliefs that stressors are uncontrollable are not motivated to cope constructively, and thus associated negative health outcomes are exacerbated [34,74]. Avoiding thinking about stressors could be an effective short-term coping strategy, as the individual may not be as preoccupied or concerned about the negative impacts or stress of shift work. However, given the limited temporary benefits associated with this strategy and the health and social consequences associated with supressing emotions [45], it may be more important for nurses to apply alternative pro-active coping strategies that address stressors in order to cope with the effects of shift work in the long-term. Additionally, many of the aforementioned themes and sub-themes were related to time management practices, underlining the value of pre-planning and organising time to engage in other coping mechanisms [68]. Pre-planning meals and allocating time to spend participating in social and leisure activities is consistently found to effectively reduce work-related stress and foster wellbeing amongst nurses [33,42].

In the fourth theme—work-related coping—the role of the workplace to facilitate breaks, rostering, workload, and supervision and debriefing coping strategies featured prominently across participants’ responses. Control over breaks, rostering, and workload acted as coping strategies because they allowed shift workers time to relax and recover. These findings correspond with previous studies that suggest that control or influence over work schedules and conditions can help manage feelings of helpless and powerless over circumstances, in turn reducing stress and improving job satisfaction [45,64,68,75]. The importance of breaks has been well established, and also allows shift workers time to engage in other coping practices, such as healthy eating and relaxation, which otherwise may not be practiced because of high workloads and stress [68]. Likewise, perceptions of greater workloads and a lack of workplace support have been linked to higher rates of job stress and reduced mental health [33,40,52]. As suggested by the nurses in the current study, allowing flexibility in work schedules and ensuring nurses take breaks so they have opportunities to rest and eat, can help nurses to balance shift work demands and other needs [58,68].

This study’s findings that supervision and debriefing are important in dealing with stressors is consistent with other studies [45,51,76,77]. Such studies have indicated that supervision and, in particular, more informal debriefing practices, helped nurses to de-stress, relax, and ultimately improved their work satisfaction and perceptions of support [76,77]. Given the importance of support from supervisors and colleagues, providing effective leadership and support networks within workplaces emerges as a critical area for improvement in order to reduce stress and other negative health outcomes associated with shift work [77].

The present study provides insight into the types of individual strategies and workplace support systems that act as mechanisms through which nurses cope with the demands associated with their job and with shift work. The large number and length of open-ended responses provided (up to a paragraph in some instances) speaks to the high level of participant engagement and value of findings in terms of the large amount of relatively rich insights generated. Given that coping strategies were found to be highly interconnected, health or wellbeing promotion initiatives that attempt to promote coping in one area in isolation from others may have unintended consequences. For instance, an initiative aimed at promoting sleep may inadvertently reduce opportunities for shift-working nurses to engage in other coping strategies such as socialising, leisure, and physical activity. Thus, future health or wellbeing promotion initiatives may benefit from a balanced approach that focuses on multiple coping strategies.

It is important to note that this study was conducted across one health service. Considering that shift work schedules and workloads may differ between organisations, findings may not be generalisable beyond this health service. However, the study is still able to provide an indication of possible coping strategies employed by nurses in Australian health settings. The current study did not examine how effective nurses perceive coping strategies to be or how they impacted levels of stress and health outcomes. Additionally, the study did not capture nurses’ full work histories, which may have influenced perceptions of stressors and adverse effects, as well as the types of coping strategies employed. Given that not all nurses in the organisation completed the survey, the sample may not be representative of all nursers at the organisation. Nurses that self-selected to complete the survey may have been more likely to experience lower job satisfaction, greater stress, and adverse health effects.

## 5. Conclusions

There is currently little knowledge on the coping mechanisms used by nurses to manage the stress and adverse effects of a shift working schedule. This study provides important insights into the range of individual coping strategies and organisational supports used by shift working nurses, highlighting opportunities to promote constructive coping strategies in order to foster wellbeing. Individual coping strategy themes supported the promotion of the benefits of social support, hobbies, time management, mindfulness, exercise, diet, and sleep practices, while increasing awareness of the possible dangers of social isolation and overreliance on substance use. Findings also suggest that, rather than simply improving nurses’ abilities to employ effective coping strategies, workplaces also play a key role in facilitating nurses’ wellbeing. The importance of organisational support highlights the need for workplaces to consider shift workers’ preferences and needs, facilitating schedule requests and effective support networks. Given that shift work is vital for health services and the work schedule of nurses, it is important for nurses to adopt effective coping strategies to help handle the high demands associated with the job, as well as in handling the adverse effects (e.g., excessive sleepiness and possible psychological distress) of a shift working schedule, in order to foster wellbeing and ultimately sustainability in the profession.

## Figures and Tables

**Table 1:** Sociodemographic and occupational characteristics of shift working nurses.

Characteristic	*N* (%) ^1^
**Gender**	
Female	394 (87.8)
Male	53 (11.8)
Non-binary/third gender	1 (0.2)
Prefer not to say	1 (0.2)
**Age**	
≤30 years	110 (28.9)
31–39 years	70 (18.4)
40–49 years	80 (21.0)
50–59 years	98 (25.7)
≥60 years	23 (6.0)
**Employment status**	
Ongoing	392 (87.3)
Fixed term	31 (6.9)
Casual	24 (5.3)
Executive contract	2 (0.4)
**Average hours worked (past month)**	
0	4 (0.9)
1–40	392 (87.3)
>40	53 (11.8)
**Years worked in current role**	
≤5 years	207 (47.9)
6–10 years	107 (24.8)
11–15 years	50 (11.6)
16–20 years	28 (6.5)
>20 years	40 (9.3)
**Types of shift worked in past month**	
Day (6:00am–7:00pm)	385 (85.7)
Evening (3:00pm–12:00am)	387 (86.2)
Night (10:00pm–8:00am)	288 (64.1)
**Frequency of night shifts (10:00pm–8:00am)**	
Nearly every day	12 (2.7)
2–4 times per week	107 (23.8)
3–4 times per month	108 (24.1)
1–2 times per month	68 (15.1)
Rarely/nearly never	153 (34.1)
**Frequency of rotating shifts**	
Nearly every day	188 (41.9)
2–4 times per week	110 (24.5)
3–4 times per month	41 (9.1)
1–2 times per month	42 (9.4)
Rarely/nearly never	68 (15.1)

^1^ Calculated according to the percentage of the valid count.
